# Preparation, Characterization and Evaluation of Antibacterial Properties of Polylactide-Polyethylene Glycol-Chitosan Active Composite Films

**DOI:** 10.3390/polym14112266

**Published:** 2022-06-01

**Authors:** Rómulo Salazar, Veronica Salas-Gomez, Adriana A. Alvarado, Haci Baykara

**Affiliations:** 1 Escuela Superior Politécnica del Litoral, ESPOL, Facultad de Ingeniería en Mecánica y Ciencias de la Producción, Campus Gustavo Galindo, Km 30.5 Vía Perimetral, P.O. BOX 09-01-5863, Guayaquil 090902, Ecuador; verdesal@espol.edu.ec; 2 Escuela Superior Politécnica del Litoral, ESPOL, Facultad de Ciencias Naturales y Matemáticas, Departamento de Química y Ciencias Ambientales, Campus Gustavo Galindo, Km 30.5 Vía Perimetral, Guayaquil 090902, Ecuador; addealva@espol.edu.ec; 3 Escuela Superior Politécnica del Litoral, ESPOL, Center of Nanotechnology Research and Development (CIDNA), Campus Gustavo Galindo, Km 30.5 Vía Perimetral, P.O. BOX 09-01-5863, Guayaquil 090902, Ecuador

**Keywords:** polylactide (PLA), chitosan, solvent casting, biodegradable films, antibacterial properties

## Abstract

Chitin is a natural biopolymer obtained from the exoskeleton of crustaceans. Chitosan is a derivative of chitin, which has antimicrobial properties and potential applications in several industries. Moreover, the composites of chitosan with other biodegradable polymers, such as polylactide (PLA) as packaging film, have shown promising results. In this study, chitosan was obtained and characterized from shrimp shells. Then, polylactide-chitosan composite films were prepared by a solvent casting technique using various amounts of chitosan (0.5–2% *w*/*w*) and polyethylene glycol as plasticizer (10% *w*/*w*). Thermal, mechanical properties, Fourier-transform infrared, scanning electron microscopy, as well as antibacterial properties of composite films were determined. It was found that adding chitosan (CH) into PLA films has a significant effect on tensile strength and no effect on thermal properties. The results showed a reduction on average of 1 log of colony-forming units against *Staphylococcus aureus*, while there is no antibacterial effect against *Salmonella typhimurium*. The study proved the antibacterial effect of CH in films of PLA against Gram-positive bacteria and appropriate mechanical properties. These films could be used for the development of biodegradable/eco-friendly food packaging prototypes, as a potential solution to replace conventional non-degradable packaging materials.

## 1. Introduction

Since the beginning of this century, in response to the social demand for reducing the environmental impact of plastic materials, new biodegradable and/or bio-based polymers have appeared as an alternative to non-degradable synthetic polymers. Consequently, the scientific literature has boosted these materials and their applications, which are oriented to various sectors, including food packaging. One of the most promising thermoplastic biodegradable materials for the packaging industry is polylactide (PLA). It can be processed using available production technologies, however, some of its properties must be improved to maintain food quality and safety. In this regard, the development of packaging materials from biodegradable renewable origin has been drawing attention worldwide and is a priority research area from an environmental point of view [1].

Polylactide (PLA) is a polyester produced from lactic acid (LA) obtained from the fermentation of corn starch and other polysaccharide sources. It is a material with significant potential for use in food packaging, as it produces highly transparent films, has good mechanical and barrier properties, and is easy to process [2,3].

On the other hand, chitin is an abundant natural biopolymer found mainly in the exoskeleton of crustaceans, which has a chemical structure of poly(β-(1-4)-2-acetamide-D-glucose. Deacetylation of chitin in an alkaline medium yields a copolymer, chitosan (CH), which contains β-(1-4)-2-acetamide-D-glucose and β-(1-4)-2-amino-D-glucose repeating units. The importance of chitosan lies in its antimicrobial properties, along with its polycationic structure and film-forming properties [4,5]. Chitosan has great potential for a wide range of applications due to its biodegradability, biocompatibility, antimicrobial activity, non-toxicity and versatile chemical and physical properties. Chitosan and its composites with neutral biopolymer can be used to prepare packaging materials and edible protective coatings for food preservation. Furthermore, chitosan can be transformed into fibers, films, gels, sponges, beads or nanoparticles [4,6,7,8].

Antimicrobial packaging materials are the most promising active packaging systems. These systems can eliminate or inhibit the spoilage of pathogenic microorganisms that contaminate food. Over the past decade, there has been a great effort to develop antimicrobial films to improve the safety and shelf life of food [9,10]. Several papers reported the preparation and potential application of PLA-chitosan composites that could be used for the aforementioned purposes [4,11,12,13].

Rapa et al. studied the effect of variation of chitosan (CH) in PLA matrices biocomposites plasticized with tributyl acetyl citrate (ATBC) on antifungal/antimicrobial activities. The authors reported that the plasticizer reduced the fracturability of PLA and that the obtained films showed improved mechanical and thermal properties. Moreover, the composite films showed significant antimicrobial activity against *S. aureus* and *E. coli* on the contact surfaces [14].

Bonilla et al. prepared films based on polylactic acid (PLA) and different amounts of chitosan (CH) by extrusion. The authors investigated the effects of the amount and CH particle size (715 and 180 μm) incorporated into the PLA matrix (5% or 10% on a PLA basis) on physicochemical characteristics and antimicrobial activity of the films. The incorporation of CH particles led to less stiffness and elasticity of the films. Additionally, the PLA/CH composites showed significant antimicrobial activity against aerobic microorganisms and total coliforms, especially with a particle size of 180 μm [12].

In the field of active packaging, several studies have been conducted to produce PLA-based composite films or systems with CH by solvent casting processing. This method is usually used as a model process due to the easy application, the good quality of the elaborated films and its suitability for processing thermosensitive polymers and active agents [15,16]. PLA films prepared by solvent casting are transparent, more flexible, and present a lower tensile strength compared to the ones formed by the other processing methods [15]. Furthermore, this method avoids the thermal degradation of chitosan, preserving its functionality and antimicrobial properties [8]. 

To the best of our knowledge this is one of the studies in Ecuador that considers added value to shrimp shells for chitosan preparation and its potential application in active non-migrating packaging film synthesis. The aim of the study is to prepare, characterize and evaluate the antimicrobial properties of PLA-chitosan composites using low concentrations of CH (0.5, 1, and 2% *w*/*w*) and polyethylene glycol as a plasticizer. The solvent casting method has been chosen for the sample preparation due to its simplicity to be used at the laboratory scale. All the samples have been characterized using thermal, mechanical properties, Fourier-transform infrared, and scanning electron microscopy. Antibacterial properties have been assessed against *Staphylococcus aureus* and *Salmonella typhimurium* by the pour-plate method. 

## 2. Materials and Methods

### 2.1. Materials

Polylactide or PLA granular Ingeo™ Biopolymer 2003D (average molecular weight is around 1.80–2.23 × 10^5^ Da) [17,18] was supplied by Nature Works^®^ Co. LLC, (Blair, NE, USA). Polyethylene glycol (PEG 300, Sigma Aldrich, Steinheim, Germany), as one of the most well-known plasticizers [19,20], and chloroform grade solvent (>99%, Merck, Darmstadt, Germany) were used for the composites’ preparation.

In addition, commercial reagent-grade chitosan (with an average molecular weight in the range of 3.10–3.75 × 10^5^ Da, deacetylation percentage of >75%, Sigma Aldrich, Reykjavík, Iceland) as a reference, and chitosan produced in the laboratory from shrimp shells (deacetylation percentage of 82%) for comparative purposes were also used. 

Tryptic Soy Broth (TSB) and Buffered Peptone Water (BPW) were purchased from Merck (Darmstadt, Germany). Baird Parker and Heart-Brain Infusion agars were purchased by Acumedia from the Neogen Corporation (Lansing, MI, USA). Reagent-grade sodium chloride was purchased from Scharlau (Barcelona, Spain). The bacterial strains used were *Staphylococcus aureus* (ATCC No. 12600) and *Salmonella typhimurium* (ATCC No. 14028), from Microbiologics (St. Cloud, MN, USA).

### 2.2. Methods

#### 2.2.1. Preparation of PLA—Chitosan Films by Solvent Casting

Chitosan has been obtained from shrimp shells according to the methodologies reported in the literature [21,22].

The composite films have been prepared according to the method reported by Rhim [15]. For this purpose, the films with different chitosan contents (0.5%, 1% and 2%) were prepared using both commercial and synthesized chitosans. A PLA solution (2% *w*/*v*) was prepared using chloroform as the solvent and kept under constant stirring for 2 h. Then, 10% (regarding PLA mass) of PEG-300, as the plasticizing agent, and chitosan (0.5%, 1% and 2%, regarding PLA mass) were added and mixed with the PLA solution until an optimal dispersion was yielded. These formulations were coded, as shown in Table 1. Afterwards, the solution was poured into glass Petri dishes, which covered the entire surface and formed a uniform layer. The samples were kept at room temperature, for at least 20 h until the solvent evaporated completely, to remove the films from the plates without any damage. All the films were stored in a desiccator until they were analyzed and characterized.

#### 2.2.2. Thermogravimetric Analysis (TGA)

The weight loss, as a function of temperature, was measured using a TA Instruments Thermal Analysis SDT model Q600 thermogravimetric analyzer (TA Instruments, New Castle, DE, USA). The samples were analyzed under a nitrogen atmosphere with a flow rate of 100 mL/min and in a temperature range from room temperature to 900 °C at a 20 °C/min heating rate.

#### 2.2.3. Differential Scanning Calorimetry (DSC)

The thermal properties of the films were determined in duplicate using a DSC Q200 (TA Instruments, New Castle, DE, USA) differential scanning calorimeter. For this technique, the film samples were analyzed under a nitrogen atmosphere with a flow rate of 40 mL/min from −30 to 200 °C at a heating ramp of 10 °C/min, with double scanning. For the calculation of the degree of crystallinity of the samples (*X_c_*), the following equation was used:

$$X_{C}=X_{PLA}\;\times \;\frac{\Delta H_{m}-\Delta H_{c}}{\Delta {H{}^{\circ}}_{m}}$$

where *X_PLA_* is the mass fraction of PLA; Δ*H_m_*, Δ*H_c_* and Δ*H°_m_* are the enthalpies of fusion, cold crystallization and fusion of crystalline PLA (93 J/g) [3,23], respectively.

#### 2.2.4. Mechanical Strength 

Mechanical properties of the films and their die-cut were done according to the ASTM D-882 norm [24]. The Shimadzu^®^ UTM-600KN universal testing equipment (Shimadzu Co., Kyoto, Japan) was used to perform tensile tests on the PLA/PEG/chitosan films at a rate of 5 mm/min. The results of the triplicated samples were recorded for maximum stress, strain at break and the modulus of elasticity.

#### 2.2.5. Fourier-Transform Infrared Spectrophotometry (FTIR)

The FTIR of the samples was carried out using a Perkin Elmer Spectrum 100 spectrophotometer (Perkin Elmer Inc., Shelton, CT, USA). The analysis was performed in the range of 4500–450 cm^−1^ with 10 consecutive scans at a resolution of 4 cm^−1^.

#### 2.2.6. Scanning Electron Microscope (SEM)

The morphological analysis of the samples was carried out using an Inspect FEI 200 model electron microscope (FEI Company, Hillsboro, OR, USA) with an accelerating voltage of 10.50 kV. For this purpose, the samples were placed on a carbon paper strip sample holder and coated with platinum to obtain images with high resolutions. 

#### 2.2.7. Evaluation of the Antimicrobial Activity of the Film Samples

The antimicrobial evaluation was performed according to a liquid culture methodology adapted from the study reported by Ahmed et al. [11,25]. The films were cut in a rectangular shape with a width of 1 cm and approximately 0.4 g of weight. Prior to the analysis, the surface of the films was disinfected with UV light for 20 s. Afterwards, the bacterial stem cell strain grew in tryptic soy broth (TSB) in incubation at 37 °C for 24 h. Then, the McFarland standard was used to obtain an approximate concentration of 1.5 × 10^8^ CFU/mL. This stock solution was diluted by peptone—water solution (25 g peptone/1 L water) to achieve the desired bacterial concentration of 10^5^ for *Staphylococcus aureus* and *Salmonella typhimurium*. The antimicrobial activity of the films was tested in duplicate against the microorganisms mentioned. 

Each film of the PLA-PG-CH previously sterilized sample was immersed in a test tube with 10 mL of TSB and 0.1 mL of the microorganism (1 × 10^5^ CFU/mL). Samples were incubated at 37 °C for 24 h. Then, a serial dilution of bacteria interacted with films was done using 0.9% sodium chloride solution. Afterwards, the diluted samples were placed in Petri dishes and inoculated with Baird’s Parker Agar for *S. aureus* and BHI for *S. typhimurium*, and incubated for 24 h at 37 °C. At the end of the incubation period, the colony-forming units were counted on each of the plates. The colony-forming unit (CFU/mL) was calculated by multiplying the average value of colony-forming units counted in each Petri dish by the dilution factor and divided by the inoculated volume (mL) of the microorganism. Finally, the quantitative reduction in bacteria on the samples was calculated using the following equation:

R = log (B/A)

where A and B are the average number of viable cells on the test sample and control sample, respectively, after 24 h incubation at 37 °C [26].

#### 2.2.8. Statistical Analysis

Statistical analysis of the data was performed by simple analysis of variance (ANOVA) using Statgraphics Centurion software (The Plains, VA, USA). Duncan’s test was used to assess the significant differences between pairs of groups at a level of *p* < 0.05. 

## 3. Results and Discussion

### 3.1. Thermogravimetric Analysis (TGA)

Figure S1 (in the Supplementary Materials file) shows the thermal properties of the films analyzed by TGA. The weight loss of the samples with commercial and synthesized chitosan is shown in Figure S1a,b.

Thermal degradation of pure PLA and chitosan has been reported as ca. 300 °C [27] and ca. 220 °C [28]. The results show that all the samples analyzed have decomposed in a single step. It was observed that PLA-PG presented two weight losses (see Figure S1c,d). The first one was presented at around 77 °C due to the elimination of the solvent trapped in the film during the casting process. The second thermal event was around 332 °C, which is attributed to the degradation of the composite materials. It is reported that the terminal hydroxyl and carboxyl groups in the PLA chains decrease the thermal stability of the polymer [29]. Furthermore, the incorporation of PEG in the PLA matrix reduces the thermal stability as well, as reported by Atiqah et al. The results obtained in the current study are consistent with the literature [29].

On the other hand, PLA-PG-F and PLA-PG-C (see Table 1) series’ showed two weight losses in their thermograms, similarly to PLA-PG. In the first thermal event (100–130 °C), the slight weight loss is attributed to the evaporation of residual water and the excess solvent entrapped in the film matrix. Residual water of the composite films could be due to the absorption of moisture from the environment by the hydroxyl groups present in chitosan. The phenomenon of water absorption has been reported by Wongphan et al., even though the composite matrix was starch [30]. Furthermore, the second thermal event (215–420 °C) represents the degradation of all the components (polylactide, polyethylene glycol and chitosan) that form the composites. This is confirmed by Peesan et al., who studied the effect of casting solvent in the elaboration of PLA/CH films, considering different percentages of CH. They indicated that the degradation temperature of both components is close to the degradation temperature of pure CH [31], which is consistent with the results presented in the current study.

Martel-Estrada et al. studied the thermal properties of poly (DL-lactide-co-glycolide)-chitosan composites. The authors reported the first thermal event between 100–150 °C, which is due to evaporation of the water content absorbed by chitosan in the composite matrices. Moreover, the authors have stated that the more chitosan content (30, 50 and 70%) in composites, the more water is evaporated in the temperature range mentioned [32]. In the current study, we have not observed the same tendency in water evaporation at low chitosan concentrations in the composites. This is probably due to the well-dried PLA and chitosan prior to film preparation.

Table 2 and Figure S2 present the results of T_max_, T_01_, T_05_ and T_09_, which represent the parameters for evaluating the thermal stability of the films. The samples show T_max_ values in the range of 317–337 °C. These indicator temperatures mean 10%, 50% and 90% of weight loss. The variation in these temperatures might be attributed to chitosan content in the films. Similar findings have been reported by Bumbudsanpharoke et al. and Han et al., considering a possible change in morphology of the polymer matrix affected by filler [33,34].

### 3.2. Differential Scanning Calorimetry (DSC)

Table 3 and Figure S3 (in the Supplementary Materials file) show the DSC results of the samples with commercial and synthesized chitosan. The pure PLA film presented a glass transition temperature (Tg) of 59.78 °C, which is in agreement with previous studies [3,35]. PLA-PG and all composites presented significantly different values between 34.67 and 37.31 °C, compared to pure PLA samples. The reduction in Tg of the composite samples can be attributed to the PEG plasticizing effect, as has been reported in the literature [2,26].

The Tg values of the PL-PG film and the PLA/CH samples presented no significant differences. Apparently, 0.5–2% of chitosan added to the composite matrix did not make any changes, neither in Tg nor in the crystallinity index, which is consistent with the literature [32]. Most of the studies in the literature reported the amount of chitosan used in composite preparations as more than 5% [12,36,37]. Those studies presented thermal properties affected by the concentration of chitosan. The values presented in Table 3 for Tc of around 115 °C and 88 °C are cold crystallization temperatures for pure PLA and composites, respectively. The reduction in Tc for composites is due to the plasticizing effect of PEG 300 [2,26].

Figure S3 (in the Supplementary Materials file) presents a double melting point which is related to different crystallites of PLA, as reported in the literature [12,14]. Considering with the melting points obtained for the composites, it is evident that the incorporation of CH in the PLA matrix has a minimal effect on the melting and crystallization behavior. This is attributed to the least interaction between the CH and PLA chain, as reported by Han et al. [34]. Hence, up to 2% of chitosan used for the composite preparation did not cause an important change in the thermal properties in general.

Table 3 shows the crystallization index (Xc) of the composites and the pure PLA. The results did not present significant differences in crystallinity by means of statistical analysis (*p* < 0.05, Duncan). The slight increase in Xc for the composites could be attributed to the effect of PEG on the PLA chains that affect the crystallization [19,26].

### 3.3. Mechanical Strength

Table 4 displays the tensile test data for each of the films incorporated with commercial and synthesized chitosan, describing the mean stress, mean strain and modulus of elasticity.

After examining the data obtained, it can be determined that the PLA-PG sample presents the highest mean stress and the highest elasticity modulus, with 24.27 MPa and 3050 MPa, respectively (see Table 4).

When analyzing the maximum stress property of the films with chitosan, it is observed that the values obtained do not have significant differences. However, they are different from the PLA-PG control sample. This could be attributed to the addition of CH in PLA-PG films that produces a decrease in the maximum stress due to the least interactions between the CH particles and the PLA matrix. This affects the stretching of the film by a weakness in the adhesion, which is reported by Fathima et al., who studied the tensile properties of PLA/chitosan films at different concentrations. The authors indicated that the increase in the amount of chitosan can form aggregates inside the film, which could be the reason for the composites’ fragility compared to the PLA-PG films [38]. On the other hand, the decrease in tensile strength, with the addition of chitosan, is due to the lack of compatibility between the films and the generation of discontinuities in the PLA matrix. Hence, it could be due to the decrease in overall cohesive forces in the matrix that makes it more sensitive to flow and breakage [12]. Additionally, non-homogeneity of the fillers inside the matrices can reduce the mechanical strength of the composites [39].

However, the films that had the greatest deformation at breakage were the films composed of PLA-PG-50F and PLA-PG, making them more flexible than the others, where they do not break by applying loads. The same phenomenon has been reported by Fathima et al., indicating the addition of a component that is not miscible in the matrix causes a decrease in the elongation properties at breakage. Similarly, the modulus of elasticity decreased in the composite samples, with respect to the PLA-PG control sample, is due to the incorporation of chitosan particles in the PLA matrix [38]. Moreover, the PLA-PG sample, which does not contain chitosan showed the highest mean strain at the break due to the plasticizer used.

### 3.4. Fourier-Transform Infrared Spectrophotometry (FTIR)

**Figure 1:**
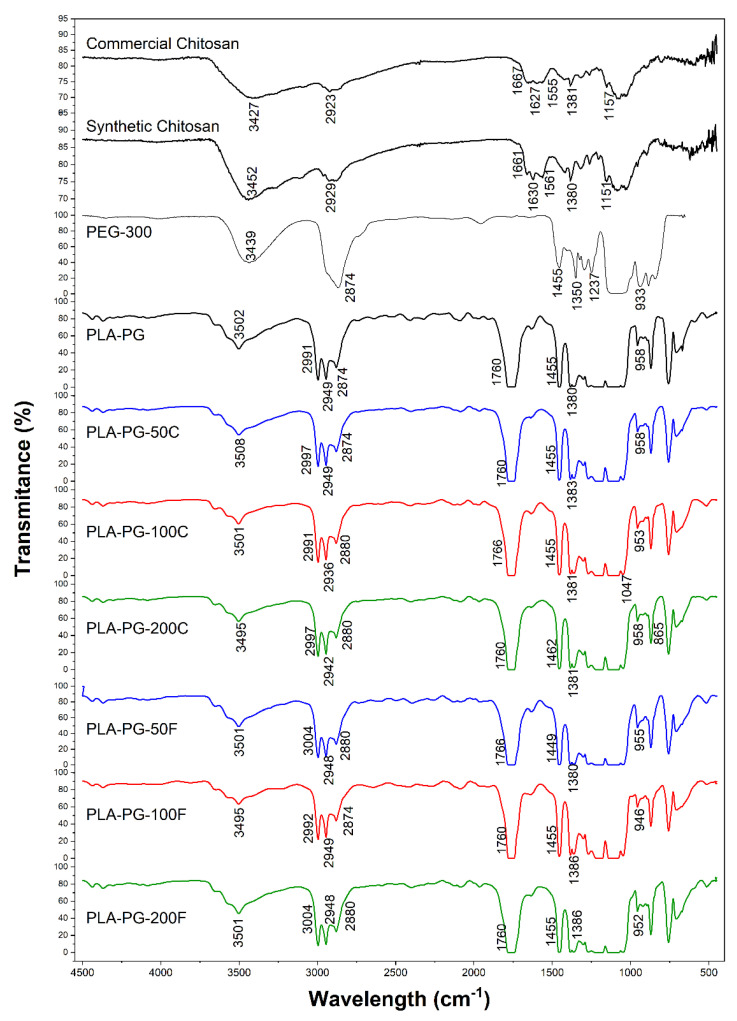
FTIR spectra of commercial and synthetic chitosan, PEG300, PLA-PG film and their different formulations.

In the FTIR spectrum of the PLA-PG sample in Figure 1 the presence of the main functional groups that characterize the polymers is observed. A strong peak at 1761.5 cm^−1^ has been observed, which is related to the stretching of the C=O vibration. The absorption band assigned to the C–H deformation vibration is detected at 1455.77 cm^−1^ and 1383.28 cm^−1^. Additionally, the asymmetric stretching vibration of -C-O-C in the PLA structure is found at 1108 cm^−1^. The symmetric and asymmetric -CH_3_ vibration, -CH stretching and C–C stretching vibration was observed at 1047.23 cm^−1^, 955.34 cm^−1^, and 870.18 cm^−1^, respectively. Other characteristic bands corresponding to the C–H stretching vibration of CH_3_ (methyl group in PLA structure) were found between 2995.7–2945.2 cm^−1^. On the other hand, the 1268.1 cm^−1^ band shows the bending of the C=O [14,34].

The characteristic peak for O–H stretching existing in PLA, PEG and chitosan structures was observed at around 3502 cm^−1^. In addition, the peaks between 3000–2879 cm^−1^ prove aliphatic C–H stretching present in the structures of the polymers [40].

As seen in Figure 1, the composites based on both synthetic and commercial chitosan showed identical FTIR spectra. On the other hand, no characteristic bands of chitosan could be identified, probably due to its low concentration used in the PLA-PG film preparation.

### 3.5. Scanning Electron Microscope (SEM)

**Figure 2:**
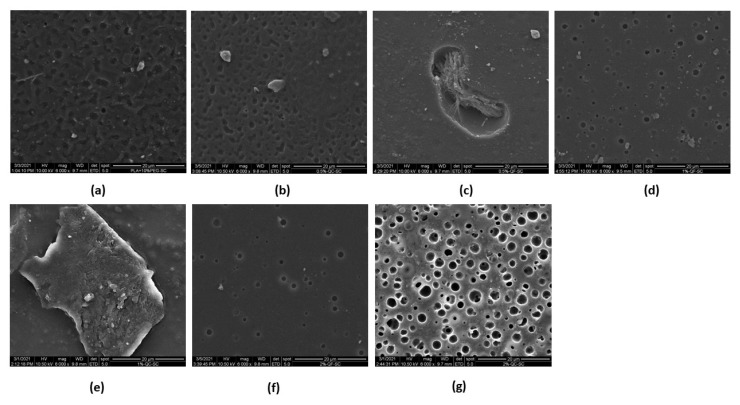
Image of the films (**a**) PLA-PG; (**b**) PLA-PG-50C; (**c**) PLA-PG-50F; (**d**) PLA-PG-100F; (**e**) PLA-PG-100C; (**f**) PLA-PG-200F and (**g**) PLA-PG-200C.

Figure 2a–g shows different SEM images that provide the surface morphology of the PLA-PG films for each of the formulations prepared with commercial and synthetic CH. In almost all the images, small porosities on the surface and a slight layer of flakes are visualized. Additionally, in some areas of the images, the fibrillar structure can be distinguished, evidencing the presence of pure CH on the surface of the PLA-PG film, similar to what has been reported by several authors [41,42]. 

In Figure 2a, it can be observed that the PLA-PG film presents cavities on the surface, which suggests that the evaporation of the solvent produces these microcavities. Peesan et al. studied the effect of the solvent casting technique on films with mixtures of hexanoyl chitosan and polylactide (20/80 and 40/60) using two different solvents (chloroform and dichloromethane). The authors have reported the same voids and porous structure formation due to the evaporation of the solvents [31]. 

On the other hand, in Figure 2e, it can be easily observed on the surface some particles that could be CH in the PLA-PG film. In Figure 2g, the SEM image of PLA-PG-200C is visualized, which shows the increase in cavities and empty spaces, that could be attributed to the increase of CH in the PLA-PG films and to the evaporation of the solvent. Some studies reported that compatibilization could be possible by the reaction of a part of the chitosan, however, it is not enough to avoid the separation of the components [32].

### 3.6. Antimicrobial Activity

The antimicrobial evaluation of films made with PLA-PG and its different combinations by incorporating commercial and synthetic chitosan (0.5%, 1% and 2%) was performed against Gram-positive *S. aureus* and Gram-negative *S. typhimurium* bacteria. The inoculated concentration in the samples was 10^5^ CFU/cm^2^ for both microorganisms. In Table 5, results are presented for PLA films prepared with chitosan at different concentrations. Additionally, the images of bacteria colonies after interaction with film samples can be seen in Figure S4.

The results obtained show a reduction in the number of bacteria of approximately 1 log from the formulations with 1% and 2% chitosan against *S. aureus* bacteria, while the results obtained for the films against *S. typhimurium* showed no antimicrobial effect. Additionally, PLA alone and PLA-PG films did not present a reduction in the number of bacteria, which is consistent with the reports in the literature [26,38]. It has been reported in the literature that chitosan has more antibacterial sensitivity against Gram-positive bacteria compared to Gram-negative ones [43]. This is consistent with the results that have been obtained in the current study. Hence, the films prepared could be used against Gram-positive bacteria, as they showed antibacterial activity against S. *aureus*. The antimicrobial effect of PLA-PG films with chitosan is due to the fact that the CH molecule possesses a positively charged amino group. This functional group interacts with anionic components, such as N-acetyluramic acid, sialic acid and neuraminic acid on the negatively charged cell surface, causing leakage of the intracellular contents of the bacteria, as reported in the literature [44,45]. In addition, several studies reported that the antimicrobial effect of CH depends on some factors, such as molecular weight, concentration and its physical form [38,45,46,47,48]. Different composite formulations can modify the film morphology and predominancy of the functional groups that affect antimicrobial properties evaluated [49].

The bactericidal properties of chitosan have been previously confirmed and reported in the literature at concentrations higher than those used in the present study. Zivanovic et al. documented the antimicrobial effect of chitosan against different foodborne microorganisms from several studies performed, such as *Salmonella enteritidis*, *Staphylococcus aureus*, *Escherichia coli* and *Lactobacillius fructivorans*. They reported the minimum inhibitory concentrations for both bacteria and yeasts, depending on the characteristics of the polymer, pH, temperature and the presence of interfering substances, such as proteins and fats. Furthermore, they expressed those variations among genera, species and strains, as well as between the same strains under different environmental conditions, which should be considered. For example, *Staphylococcus aureus* and *Salmonella typhimurium* presented bacterial inhibition from 0.005–1% and 0.06–0.2%, respectively [48].

Ardila et al. investigated the antibacterial activity of chitosan powder and flakes against three different bacterial species (*Escherichia coli*, *Listeria innocua* and *Staphylococcus aureus*) by measuring the effect of the CH concentration between 0.01 and 4% (*w*/*v*). They observed that, upon suspension in phosphate-buffered solution, the antibacterial activity of CH increases up to a certain value, which is termed critical concentration (CC). Beyond CC, the activity decreases. Additionally, the antibacterial activity of 0.4% of partially solubilized chitosan and particles in solid-state were quantified. They have demonstrated a log reduction of 4.0 and 2.5 in the bacterial density of CH solubilized in suspensions with powder and flakes, respectively. On the other hand, a reduction of 2.7 and 0.8 logs in antibacterial activity was obtained by the presence of CH in powder and flakes, favoring its solid-state antibacterial activity [47]. Additionally, the incorporation of antibacterial compounds into the biodegradable composites can help to increase the shelf life of the foodstuff in contact [50].

Rodriguez-Nuñez et al. compared the antibacterial effect of polyamide 6/66-CH films coated with CH, obtaining 0.45, 0.9, 1.13 and 1.35 mg of CH/cm^2^ of the film surface. They used the agar disk-diffusion method to determine antimicrobial activity in the films, demonstrating that the coated films exhibited antimicrobial activity against *S. typhimurium* and *S. aureus* in a range of 10–36% of the film’s surface. On the other hand, the authors have demonstrated that a 1000 and 2000 ppm CH solution has a 1–2 log reduction against *S. typhimurium* and *S. aureus* [5].

Fathima et al. observed, in PLA films with nano chitosan (0.5, 1 and 2%), a bacterial reduction percentage against *Listeria monocytogenes* (Gram-positive) and *E. coli* (Gram-negative) between 2.23–67.09% and 5.06–30.46%, respectively. In addition, the authors reported a maximum bacterial reduction using 2% of nano chitosan [38]. In this study, we obtained the reduction percentages for *S. aureus* between 36–95% and 48–95% for the films containing commercial and synthetic chitosan, respectively. While for *S. typhimurium*, the results were between 10–23% and 20–67% for commercial and synthetic chitosan-based films, respectively. The results obtained in the current study, which used a similar amount of chitosan for the film preparation, showed very high inhibition compared to the literature.

On the other hand, Mania et al. elaborated on the PLA-CH blend, using polyethylene glycol plasticizer via the solvent casting method. The authors evaluated the impact of different concentrations of chitosan, such as 4, 12 and 16 wt. % against *E. coli* and *S. aureus* bacteria. They have reported that antibacterial activity is proportional to the content of chitosan in the blend. For example, the blend with 4% and 16% of chitosan showed a log reduction of 1.57 and 1.84, 2.94 and 3.08 logs for *S. aureus* and *E. coli*, respectively [26]. In our study, considering concentrations lower than those reported by Mania et al., a log reduction of 1.32 and 1.29 against *S. aureus* was obtained for commercial and synthetic chitosan, respectively. Moreover, a log reduction of 0.11 and 0.48 against *S. typhimurium* was obtained for commercial and synthetic chitosan, respectively.

## 4. Conclusions

This study evaluated the physical and mechanical properties and the antimicrobial effect of different concentrations of CH-based PLA composites. The solvent casting method is a very appropriate technique for the preparation of PLA-PG-CH composites with acceptable mechanical and thermal properties. On the other hand, micropores in the structures of the films were formed using this method. The films prepared showed antimicrobial activity against *S. aureus* for the samples with 1–2% CH concentrations, while no antimicrobial activity against *S. typhimurium* was observed for all the samples. Hence, the results obtained in the current study, even with lower concentrations of CH, are very promising for potential applications in the food packaging industry.

## Figures and Tables

**Table 1:** Coding of the formulations of the elaborated films.

Serie *	PLA (%)	PEG (%) **	Synthesized CH (%) **	Commercial CH (%) **
PLA-PG	100	10	-	-
PLA-PG-50F	100	10	0.5	-
PLA-PG-100F	100	10	1.0	-
PLA-PG-200F	100	10	2.0	-
PLA-PG-50C	100	10	-	0.5
PLA-PG-100C	100	10	-	1.0
PLA-PG-200C	100	10	-	2.0

* PLA: polylactide; PG: PEG 300; C: commercial chitosan; F: synthesized chitosan. ** Regarding the mass of PLA.

**Table 2:** Data obtained from the DTG curve for the films studied.

Serie	T_i_ (°C)	T_max_ (°C)	T_01_	T_05_	T_09_
PLA-PG	239.55	332.27	282.32	327.89	358.76
PLA-PG-50F	220.26	321.65	278.6	317.05	344.82
PLA-PG-100F	227.07	322.28	279.42	315.67	342.91
PLA-PG-200F	228.20	332.96	290.38	327.06	344.82
PLA-PG-50C	222.53	322.97	277.90	318.1	353.76
PLA-PG-100C	237.28	337.82	294.19	330.87	356.56
PLA-PG-200C	215.72	317.22	281.71	314.73	341.1

T_i_: Initial decomposition temperature; T_max_: Maximum degradation temperature; T_01_: Temperature at 10% weight loss; T_05_: Temperature at 50% weight loss; T_09_: Temperature at 90% weight loss.

**Table 3:** Thermal properties of films prepared by the solvent casting technique.

Serie	Tg (°C)	Tc (°C)	Tm (°C)	Xc (%)
PLA	59.78 ± 0.16 ^a^	115.48 ± 2.98 ^a^	149.71 ± 0.75 ^a^	1.23 ± 0.14 ^a^
PLA-PG	35.95 ± 0.72 ^b,c^	88.00 ± 0.86 ^b^	150.33 ± 0.13 ^a,b^	2.90 ± 0.66 ^a^
PLA-PG-50F	35.98 ± 0.20 ^b,c^	87.15 ± 1.18 ^b^	150.58 ± 0.01 ^a,b^	1.61 ± 0.35 ^a^
PLA-PG-100F	36.25 ± 0.94 ^b^	89.74 ± 1.48 ^b^	150.50 ± 0.41 ^a,b^	2.86 ± 1.31 ^a^
PLA-PG-200F	36.74 ± 0.35 ^b^	89.28 ± 0.29 ^b^	150.39 ± 0.35 ^a,b^	2.30 ± 0.74 ^a^
PLA-PG-50C	36.48 ± 0.04 ^b^	88.00 ± 0.02 ^b^	150.38 ± 0.27 ^a,b^	2.57 ± 0.28 ^a^
PLA-PG-100C	34.67 ± 0.91 ^c^	87.62 ± 1.77 ^b^	150.13 ± 0.75 ^a,b^	2.27 ± 0.44 ^a^
PLA-PG-200C	37.31 ± 0.57 ^b^	89.75 ± 0.14 ^b^	151.01 ± 0.10 ^b^	1.71 ± 1.04 ^a^

Tg: Glass transition temperature; Tm: Melting temperature; Xc: Degree of crystallinity. Values followed by different letters within a column indicate significant differences at *p* < 0.05 (Duncan).

**Table 4:** Mean Maximum Stress, Modulus of Elasticity and Mean Strain at Break of PLA/PEG/chitosan films.

Serie	Mean Maximum Stress (MPa)	Mean Strain at Break (%)	Modulus of Elasticity (MPa)
PLA	17.53 ± 3.09 ^a,b^	2.16 ± 0.36 ^a^	1233.33 ± 159.48 ^a^
PLA-PG	24.27 ± 4.74 ^a^	7.11 ± 3.14 ^b^	3050.00 ± 440.34 ^b^
PLA-PG-50C	13.99 ± 5.70 ^b^	3.48 ± 0.96 ^a^	1783.33 ± 698.95 ^a^
PLA-PG-100C	11.88 ± 8.91 ^b^	2.92 ± 1.20 ^a^	1406.33 ± 1168.51 ^a^
PLA-PG-200C	14.10 ± 4.29 ^b^	3.02 ± 1.06 ^a^	1773.33 ± 637.91 ^a^
PLA-PG-50F	13.63 ± 0.68 ^b^	4.46 ± 0.61 ^a^	1110.00 ± 65.57 ^a^
PLA-PG-100F	15.87 ± 2.93 ^a,b^	3.15 ± 1.04 ^a^	1946.67 ± 358.52 ^a^
PLA-PG-200F	10.99 ± 0.98 ^b^	3.29 ± 1.27 ^a^	1463.33 ± 119.30 ^a^

Values followed by different letters within a column indicate significant differences at *p* < 0.05 (Duncan).

**Table 5:** Bacterial growth after 24 h of incubation for the microorganisms *S. aureus* and *S. typhimurium* on PLA films made with chitosan.

Serie	Average Cell Number (CFU/mL)		Log Reduction	
	*S. aureus*	*S. typhimurium*	*S. aureus*	*S. typhimurium*
Control	6.42 × 10^10^	3.22 × 10^10^		
CH 1%	4.68 × 10^8^	3.05 × 10^9^	2.14 ± 0.06 ^d^	1.02 ± 0.06 ^b^
PLA	6.74 × 10^10^	2.91 × 10^10^	−0.02 ± 0.10 ^a^	0.05 ± 0.02 ^a^
PLA-PG	5.76 × 10^10^	2.33 × 10^10^	0.05 ± 0.04 ^a^	0.14 ± 0.05 ^a^
PL-PG-50F	3.33 × 10^10^	2.67 × 10^10^	0.29 ± 0.31 ^a,b^	0.05 ± 0.01 ^a^
PL-PG-100F	5.14 × 10^9^	1.04 × 10^10^	1.10 ± 0.76 ^b,c^	0.11 ± 0.09 ^a^
PL-PG-200F	3.62 × 10^9^	2.46 × 10^10^	1.29 ± 0.57 ^c,d^	0.09 ± 0.23 ^a^
PL-PG-50C	4.12 × 10^10^	2.90 × 10^10^	0.19 ± 0.03 ^a^	0.09 ± 0.32 ^a^
PL-PG-100C	3.08 × 10^9^	2.59 × 10^10^	1.32 ± 0.17 ^c,d^	0.48 ± 0.15 ^a^
PL-PG-200C	4.64 × 10^9^	2.58 × 10^10^	1.14 ± 0.43 ^b,c^	0.17 ± 0.46 ^a^

Values followed by different letters within a column indicate significant differences at *p* < 0.05 (Duncan).

## Data Availability

Not applicable.

## Supplementary Materials

The following are available at https://www.mdpi.com/article/10.3390/polym14112266/s1, Figure S1: Thermogravimetric curves of PLA-PEG samples with chitosan at different concentrations; (a) TGA synthetic chitosan; (b) TGA commercial chitosan; (c) dTGA commercial chitosan; (d) dTGA synthetic chitosan. Figure S2: Thermogravimetric curves of PLA-PEG samples with chitosan at different concentrations showing the values of T_i_, T_max_, T_01_, T_05_, and T_09_. Figure S3: DSC thermal curves of the samples: (a) PLA-PG-CH (synthetic) and (b) PLA-PG-CH (commercial). Figure S4. Microbiological essay images showing the impact of each film sample against bacteria studied: (a) PLA-PG-CH (synthetic) and (b) PLA-PG-CH (commercial).
