# Supplementary material for: Preparation, Characterization and Evaluation of Antibacterial Properties of Polylactide-Polyethylene Glycol-Chitosan Active Composite Films
Source: Polymers (Basel). 2022 Jun 1;14(11):2266. doi: 10.3390/polym14112266 (PMC9183075; doi:10.3390/polym14112266)
Supplement: Supplementary file 1 [file polymers-14-02266-s001.zip › polymers-1722798-supplementary.pdf]

Preparation, Characterization and Evaluation of Antibacterial Properties of Polylactide-Polyethylene Glycol-Chitosan Active Composite Films

Rómulo Salazar [1,*], Veronica Salas-Gomez [1], Adriana A. Alvarado [2] and Haci Baykara [1,3,*]

[1] Facultad de Ingeniería en Mecánica y Ciencias de la Producción, Escuela Superior Politécnica del Litoral—ESPOL, Campus Gustavo Galindo, Km 30.5 Vía Perimetral, Guayaquil 090902, Ecuador; verdesal@espol.edu.ec
[2] Departamento de Química y Ciencias Ambientales, Facultad de Ciencias Naturales y Matemáticas, Escuela Superior Politécnica del Litoral—ESPOL, Campus Gustavo Galindo, Km 30.5 Vía Perimetral, Guayaquil 090902, Ecuador; addealva@espol.edu.ec
[3] Center of Nanotechnology Research and Development (CIDNA), Escuela Superior Politécnica del Litoral—ESPOL, Campus Gustavo Galindo, Km 30.5 Vía Perimetral, Guayaquil 090902, Ecuador
* Correspondence: rvsalaza@espol.edu.ec (R.S.); hbaykara@espol.edu.ec (H.B.)

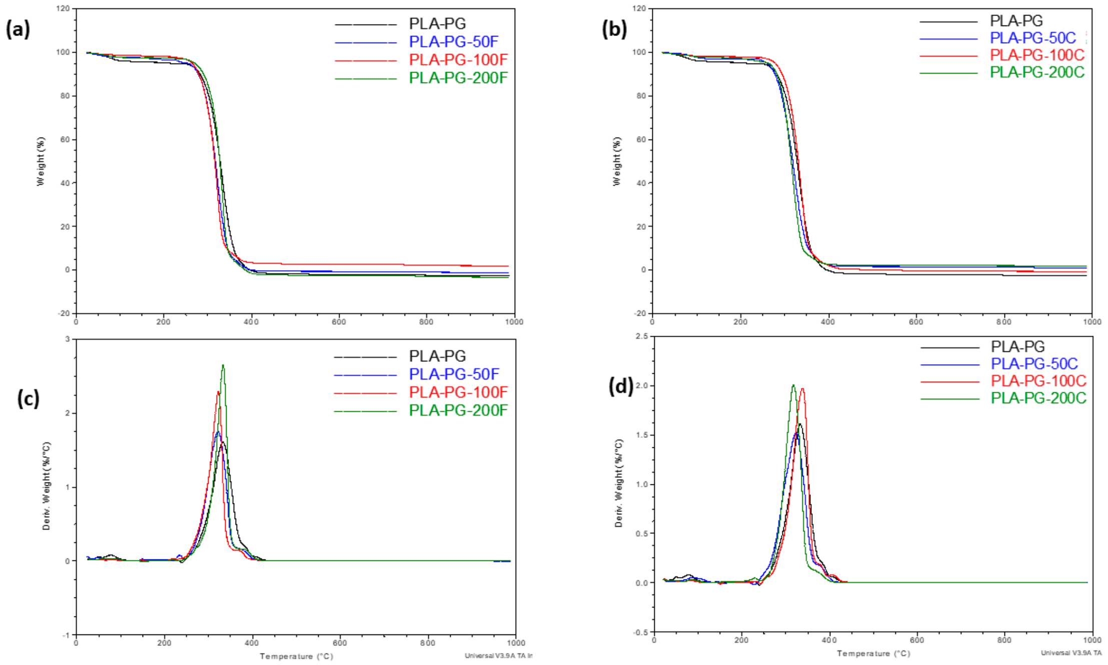

Figure S1. Thermogravimetric curves of PLA-PEG samples with chitosan at different concentrations: (a) TGA synthetic chitosan; (b) TGA commercial chitosan; (c) dTGA commercial chitosan; (d) dTGA synthetic chitosan.

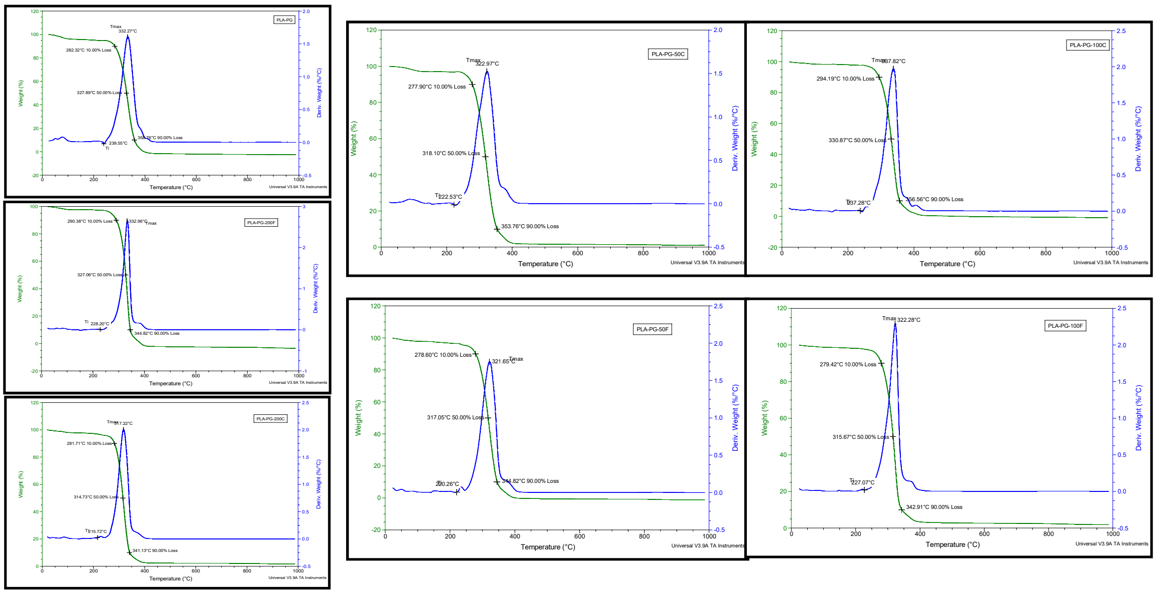

Figure S2. Thermogravimetric curves of PLA-PEG samples with chitosan at different concentrations showing the values of T_i, T_{max}, T_{01}, T_{05}, and T_{09}.

(a)

(b)

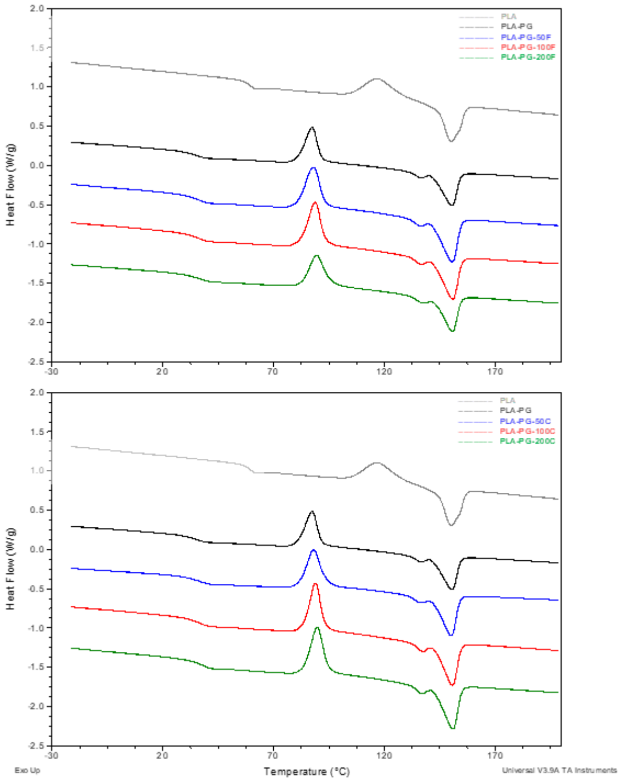

Figure S3. DSC thermal curves of the samples: (a) PLA-PG-CH (synthetic) and (b) PLA-PG-CH (commercial).

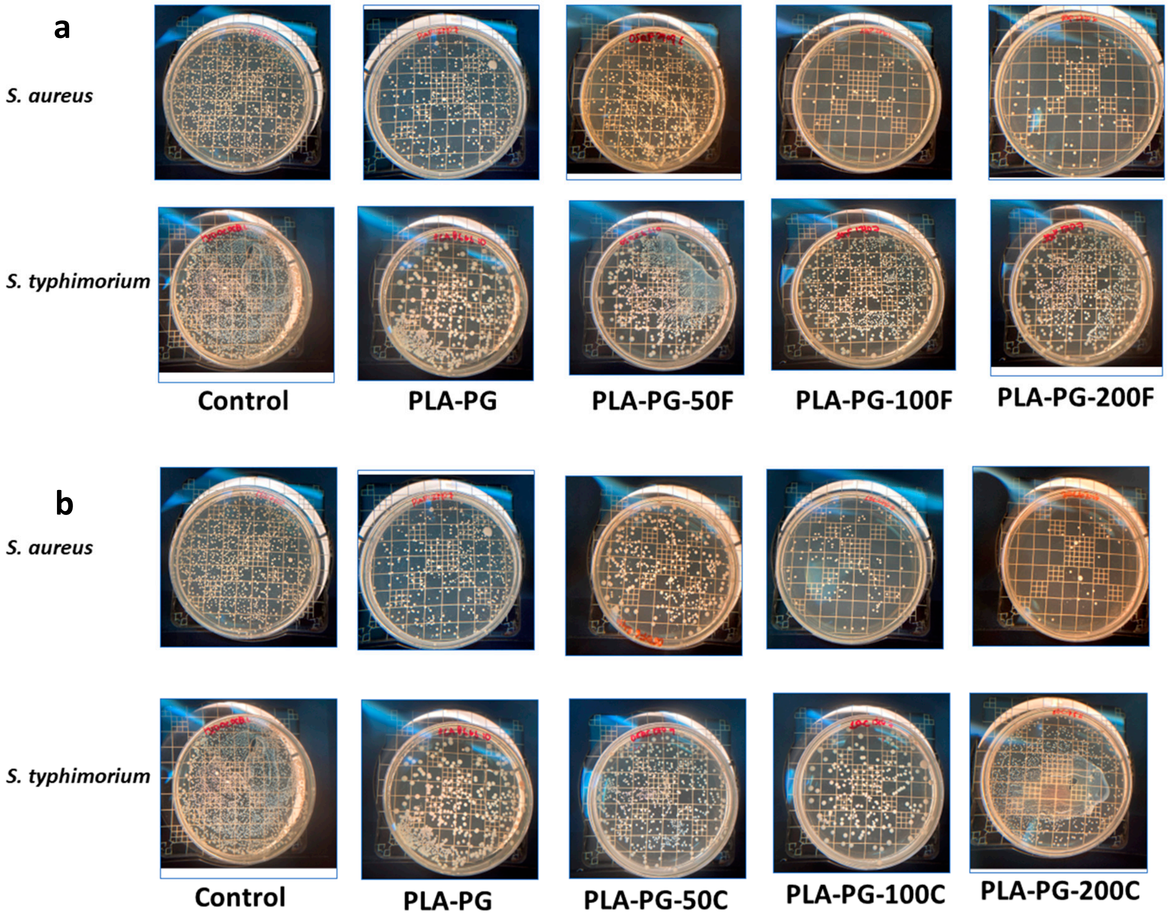

Figure S4. Microbiological essay images showing the impact of each film sample against bacteria studied: (a) PLA-PG-CH (synthetic) and (b) PLA-PG-CH (commercial).
